# Shrimp Antiviral mja-miR-35 Targets *CHI3L1* in Human M2 Macrophages and Suppresses Breast Cancer Metastasis

**DOI:** 10.3389/fimmu.2018.02071

**Published:** 2018-09-12

**Authors:** Yulei Chen, Siyuan Zhang, Jiao Cao, Xiaobo Zhang

**Affiliations:** ^1^College of Life Sciences, Zhejiang University, Hangzhou, China; ^2^Laboratory for Marine Biology and Biotechnology, Qingdao National Laboratory for Marine Science and Technology, Qingdao, China

**Keywords:** breast cancer metastasis, human anti-tumor, M2 macrophage, mja-miR-35, shrimp antivirus

## Abstract

Virus infection can change host's metabolism, while tumorigenesis results from metabolic disorder. MicroRNAs (miRNAs), crucial regulatory factors overlaying transcriptional control mechanisms, can guide metabolic homeostasis. In terms of metabolic disorder, antiviral miRNAs may have anti-tumor activity. However, this issue has not been extensively investigated. In the present study, the results revealed that shrimp mja-miR-35, which showed antiviral activity in shrimp against white spot syndrome virus (WSSV) infection, could suppress the metastasis of breast cancer by targeting human *CHI3L1* gene of M2 macrophages in a cross-phylum manner. Furthermore, the feed expressing shrimp mja-miR-35 had antiviral capacity in shrimp and anti-tumor activity in humans, leading to the simultaneous control of virus infection and tumor progression. Therefore, our findings indicated that the antiviral miRNAs derived from shrimp stress responses against virus infection might be an important source of human anti-tumor drugs and miRNAs could bridge the control of aquaculture diseases and the prevention of human tumors.

## Introduction

Shrimp, one of the most important species in aquaculture, is affected worldwide by diseases, especially those caused by white spot syndrome virus (WSSV) (1). During virus infection, viruses rely on the metabolic mechanisms of living cells to complete their life cycles and to disturb the normal metabolism of host cells, thus maximizing their replication and overcoming the host cell defense mechanisms (2). At the early stage of virus infection, in order to accomplish the energy requirement of viruses, viral products modulate host cell metabolic homeostasis to trigger pentose phosphate pathway, glycolysis, and fatty acid metabolism (3–6). When virion maturation is accomplished, damage to the host cell's metabolism will result in cell death, which in turn releases new virions (3–6). In shrimp, a Warburg-like effect is induced in hemocytes of WSSV-infected shrimp at the early stage of virus infection, which provides the virus with high demand for cellular energy and basic building blocks during replication (6). The WSSV-induced Warburg effect of shrimp is triggered via the PI3K-Akt-mTOR pathway (7). As reported, WSSV infection also causes absorption inhibition of amino acids and disturb protein metabolism as well as cell metabolism in favor of its replication (8). Emerging evidences indicate that impaired cellular energy metabolism is the representative characteristic of nearly all cancers in spite of cellular or tissue origin, especially aerobic glycolysis (9, 10). Virus infection can cause metabolic disorder of host, while tumor development comes from metabolic disorder. In this context, there is a relationship between virus infection and tumorigenesis on the aspect of metabolic disorder. However, this relationship has not been intensively explored.

Generally, metabolic disorder is associated with the regulation of gene expression. In the majority of cancers examined, the genes for glycolysis are overexpressed (9, 11). The glucose activity of carcinomas is manifested as the result of the arrangement of glycolysis and the deficiency of mitochondrial activity. The transcriptional activity of c-myc and the stabilization of hypoxia inducible factor 1 alpha (HIF1α) in cancer cells enhance the expressions of most glycolytic enzyme genes, including lactate dehydrogenase-A (LDHA) and GLUT1, which in turn increase tumor aggressiveness and lead to poor survival (11). As well-known, microRNAs (miRNAs) play essential roles in gene expression regulation. MiRNAs, a class of 18~25 nucleotide non-coding RNAs, predominantly combine with the 3′ UTRs (untranslated regions) of their target mRNAs, resulting in the destabilization or degradation of the mRNAs (12, 13). During virus-host interaction and tumorigenesis, miRNAs represent crucial regulatory factors overlaying transcriptional control mechanisms in guiding metabolic homeostasis (13, 14). Of the 63 host miRNAs in shrimp, 31 miRNAs are differentially expressed in shrimp in response to WSSV infection, which take great effects on virus infection (13). It is found that more than half of the miRNA genes are located in fragile sites or cancer-associated genomic sequences (14). One of the most important features of the miRNAs in cancers is their discrimitive roles for different cancer types or the correlation with specific neoplastic events, such as oncogenic activation (15). Dysregulation of miRNAs can contribute to metabolic abnormalities. It is revealed that miR-33a and miR-33b play crucial roles in controlling lipid and cholesterol metabolism by targeting their target genes, the sterol-regulatory element-binding protein transcription factors (16). MiR-103 and miR-107 regulate glucose and insulin homeostasis (17), while miR-34a is a key regulator of hepatic lipid homeostasis (18). As reported, an individual miRNA can exert its function by targeting multiple mRNAs (19, 20). In this context, the antiviral miRNAs of invertebrate may possess anti-tumor activity of humans in a cross-species manner. However, this issue is not intensively investigated.

To explore the role of antiviral miRNA in tumorigenesis, mja-miR-35, an antiviral miRNA of shrimp was characterized in the present study. As predicted, mja-miR-35 could target human *CHI3L1* gene. Our previous study reveals that M2 macrophage-secreted CHI3L1 protein promotes the metastasis of gastric and breast cancer cells by interacting with interleukin-13 receptor α2 chain (IL-13Rα2) molecules on the plasma membranes of cancer cells (21). In this context, the role of mja-miR-35 in antiviral immunity and tumor metastasis was further explored in the present investigation. The results showed that mja-miR-35 played a positive role in the shrimp antiviral immunity and could suppress breast cancer metastasis in a cross-phylum manner.

## Materials and methods

### Cell culture

The MDA-MB-231 breast cancer cell line and the THP-1 human acute monocytic leukemia cell line were purchased from The Cell Bank of The Chinese Academy of Sciences (Shanghai, China). THP-1 cells were cultured in a humidified atmosphere of 5% CO_2_ at 37°C with RPMI 1640 medium (HyClone, UT, USA) supplemented with 10% fetal bovine serum (FBS) (Gibco, Grand Island, NY, USA). MDA-MB-231 cells were grown at 37°C in a humidified atmosphere (CO_2_ was not present) with Leibovitz's L-15 medium (HyClone) supplemented with 10% FBS (Gibco).

### Prediction of the miRNAs targeting *CHI3L1*

To predict the shrimp miRNAs targeting *CHI3L1* mRNA 3′ UTR, the human *CHI3L1* mRNA sequence (GenBank accession no. NM_001276) was employed. To predict the WSSV target genes of mja-miR-35, the 3′ UTRs of WSSV genes were used. The prediction was conducted using two independent computational algorithms TargetScan 5.1 (http://www.targetscan.org) and miRanda (http://cbio.mskcc.org/microrna_data/manual.html). The results predicted by the two algorithms were combined and the overlaps were calculated.

### Polarization of THP-1 monocytes into M2 macrophages

THP-1 cells (1 × 10^6^ cells/mL) were treated with 320 nM phorbol 12-myristate 13-acetate (Sigma, St. Louis, MO, USA) for 6 h. Then the culture medium was supplemented with 20 ng/ml IL-4 (PeproTech, Rocky Hill, NJ, USA) and 20 ng/ml IL-13 (PeproTech) for M2 macrophage polarization. The cells were cultured for an additional 18 h to generate M2 macrophages.

### Expression of mja-miR-35 in M2 macrophages

For cell transfection, mja-miR-35 (5′-AACUGUGAGAAGUUCCGGUUU-3′) and mja-miR-35-scrambled (5′-UUCUCCGAACGUGUCACGUUU-3′) were synthesized by GenePharma (Shanghai, China). When the M2 macrophages were 30–50% confluent, the cells were transfected with miRNA using Lipofectamine RNAiMAX (Life Technologies, Carlsbad, CA, USA) according to the manufacturer's instructions. The final concentration of miRNA was 100 nM. At 24 h after transfection, the cells were subjected to further experiments.

### Quantitative real-time PCR detection of mRNA

Total RNAs were extracted from cells or tissues using an RNAprep Pure Cell/Bacteria kit (Tiangen, Shanghai, China). The first-strand cDNA was synthesized from the total RNA using a PrimeScript™ 1st Strand cDNA Synthesis kit (Takara Bio Inc., Shiga, Japan). Subsequently quantitative real-time PCR was conducted using gene-specific primers (*CHI3L1*, 5′-CACCATTGACAGCAGCTATGACATT-3′ and 5′-GCATCCTCCTGACCTCGGAAC-3′; human *GAPDH*, 5′-GGTATCGTGGAAGG ACTCATGAC-3′ and 5′-ATGCCAGTGAGCTTCCCGTTCAG-3′; *wsv140*, 5′-CTC CTCTACATCATCCATTTCATT-3′ and 5′-ATCGTCAATACCAGGTCTATCTAC T-3′; *wsv279*, 5′-TGTGGTGTAGTAGGGGCAGAT-3′ and 5′-CTAAAGGTTGAAG AGAAGGTGGATC-3′; *wsv309*, 5′-ATGCTAGGCGTTTTGTTATTCATG-3′ and 5′-CTATTGAAAATGTCTGTTGCTCGC-3′; *wsv361*, 5′-TCTTTGTCCCTATCTTA GCAGCG-3′ and 5′-TCGGACCCTATTTTCTCTATTGTTC-3′; shrimp *GAPDH*, 5′-GGTGCCGAGTACATCGTTGAGTC-3′ and 5′-GGCAGTTGGTAGTGCAAGA GGC-3′) to determine the expression levels of *CHI3L1, wsv140, wsv279, wsv309, wsv361* and *GAPDH* genes. SYBR Premix Ex Taq™ (Takara) was used according to the manufacturer′s instructions to quantify the transcript levels of genes. After initial denaturation at 95°C for 30 s, 50 cycles of PCR amplification were performed as following: 95°C for 5 s and 60°C for 30 s. Melt curves were then generated, and the relative level of the target gene mRNA was normalized to that of *GAPDH*.

### Western blot analysis

Cells were lysed in RIPA lysis buffer (Beyotime Institute of Biotechnology, Shanghai, China) with 2 mM phenylmethanesulfonyl fluoride (PMSF, Solarbio, Beijing, China) on ice. The proteins of the cell lysates were separated on a 10% SDS-polyacrylamide gel (SDS-PAGE) and then electrotransferred to a nitrocellulose membrane (GE Healthcare, Waukesha, WI, USA) in transferring buffer (25 mM Tris-HCl, 190 mM glycine, 20% methanol). After blocking with 5% non-fat milk in TBST buffer (20 mM Tris-HCl, 150 mM NaCl, 0.05% Tween-20, pH 8.0) for 90 min at room temperature, the membrane was incubated with a primary antibody in TBST buffer containing 1% non-fat milk overnight at 4°C. Subsequently, the membrane was incubated with a horseradish peroxidase (HRP)-conjugated secondary antibody (BioRad, Hercules, CA, USA) for 2 h at room temperature. The membrane was developed using a Western Lightning Plus-ECL kit (Perkin Elmer, Waltham, MA, USA). The CHI3L1 primary antibody was purchased from Abcam (Cambridge, MA, USA). The β-actin primary antibody was purchased from Santa Cruz Biotechnology (Santa Cruz, CA, USA).

### Wound-healing assay

MDA-MB-231 cells were grown to 80% confluence in a 24-well plate. Subsequently a linear wound was created in the confluent monolayer using a 100-μl pipette tip and the cells were washed three times with PBS (phosphate buffered saline). To assess the effects of miRNAs on cancer cell migration, M2 macrophages alone or M2 macrophages transfected with miRNAs were placed on a 0.4-μm pore-size chamber insert and were co-cultured with the cancer cells by immersion in the cancer cell culture medium. At 24 h after co-culturing, the cancer cells were examined using a phase-contrast microscope.

### Shrimp culture and WSSV infection

Shrimp (*Marsupenaeus japonicus*), approximately 10 g, were cultured in groups of 20 individuals in tanks containing 80 liters of aerated seawater at 20–25°C. To ensure that the specimens were virus-free, shrimp were detected using PCR with WSSV-specific primers (5′-TTGGTTTCATGCCCGAGATT-3′ and 5′-CCTTGGTCA GCCCCTTGA-3′) prior to experimental infection by WSSV. The healthy shrimp were infected with 100 μl of WSSV virus solution (10^5^ copies/ml) by injection into the lateral area of the fourth abdominal segment. Phosphate-buffered saline (PBS) was subjected as a control. At different times post-infection, the shrimp were collected for later use.

### Detection of mja-miR-35 by northern blotting

Total RNAs, extracted from shrimp with the mirVana™ miRNA isolation kit (Ambion, Austin, TX, USA), were separated on a denaturing 15% polyacrylamide gel with 8 M urea. Then the RNAs were transferred to a Hybond-N+ nylon membrane (Amersham Biosciences, GE Healthcare, UK), followed by UV cross-linking. Then the membrane was prehybridized in 10 ml DIG Easy Hyb granule buffer (Roche, Basel, Switzerland) for 30 min and hybridized with digoxigenin (DIG)-labeled probe completely complementary to mja-miR-35 (5′ DIG-ACCGGAACTTCTCACAGTT-3′) overnight at 42°C. The DIG-labeled U6 probe (5′ DIG-GGGCCATGCTAATCTT CTCTGTATCGTT-3′) was used as a loading control. The membrane was incubated with an AP (alkaline phosphatase)-conjugated anti-DIG IgG (Roche) for 2 h at room temperature. Immunological detection was performed using the DIG High Prime DNA labeling and detection starter kit II (Roche).

### Detection of WSSV copies by quantitative real-time PCR

The WSSV copies of shrimp were determined by quantitative real-time PCR (qPCR). DNA extracted from shrimp hemocytes using a tissue DNA extraction kit (Tiangen) was subjected to qPCR with WSSV-specific primers (5′-TTGGTTTCAG CCCGAGATT-3′ and 5′-CCTTGGTCAGCCCCTTGA-3′) and probe (5′-FAM-TGC TGCCGTCTCCAA-Eclipse-3′). A linearized plasmid containing a 1400-bp DNA fragment from the WSSV genome was quantified and serially diluted as an internal standard. The 10 μl PCR solution contained 5 μl of Premix Ex Taq (Perfect Real Time) (TaKaRa), 0.2 μl of 10 μM forward and reverse primer respectively, 0.15 μl of 10 μM probe, 200 ng of DNA template, and distilled water up to 10 μl. The PCR program was 95°C for 1 min, followed by 45 cycles of 30 s at 95°C, 30 s at 52°C, and 30 s at 72°C.

### Shrimp mortality analysis

The virus-free shrimp were intramuscularly injected with 100 μl of WSSV virus solution (10^5^ copies/ml) which was mixed with the synthesized mja-miR-35. As controls, WSSV alone and mja-miR-35-scrambled were included in the injections. Twenty shrimp were used for each treatment. The cumulative mortality of shrimp was examined every day. The experiments were biologically repeated three times.

### Prediction of target genes

To predict the target genes of mja-miR-35, the WSSV genome sequence (GenBank accession number AF332093) was employed with three independent computational algorithms including TargetScan 5.1 (http://www.targetscan.org), miRanda (http://cbio.mskcc.org/microrna_data/manual.html) and Pictar (http://pictar.mdc-berlin.de/). The overlapped genes predicted by three algorithms were considered to the potential targets of miRNA.

### Plasmid construction and fluorescence assay

The 3′ UTR of WSSV gene and enhanced green fluorescent protein (EGFP) gene were cloned into a pIZ/V5-His vector (Invitrogen, Carlsbad, CA, USA). The EGFP gene was amplified from the pEGFP vector (BD Biosciences, San Jose, CA, USA) and cloned into the pIZ/V5 vector to produce a control EGFP construct. Subsequently, the 3′ UTRs of WSSV genes was separately cloned into the pIZ/V5 vector downstream of EGFP using the Xba I and Sac II restriction sites to generate the EGFP-3′ UTR construct. The WSSV gene 3′ UTRs were cloned with gene-specific primers (wsv140, 5′-CGGGTCTAGACGTACAAAATCTATAGCTTG-3′ and 5′-CCG CGGTGAAATGGAACCTGATGGTT-3′; wsv279, 5′-GGGTCTAGAAAGGTTAG TGTACAACTCAG-3′ and 5′-AACCGCGGTGTTACACAAGCTGTTTT-3′; wsv309, 5′-TCTAGATCCACATTATCCAGTTTCCC-3′ and 5′-CCGCGGTGTTTAAAATG TGTGATATT-3′; wsv361, 5′-AATCTAGAGAAGCTGCGATTGACCT-3′ and 5′-C CGCGGACAGATGCGTAGAGT-3′). As controls, the 3′ UTR sequences complementary to the mja-miR-35 seed sequence (5′-ACUGUGAG-3′) were mutated generating the EGFP-3′ UTR-mutant constructs using sequence-specific primers (wsv140, 5′-CCCATGTGTCGTAACAGTGAGTACTAACAATTGC-3′ and 5′- GTG CAATTGTTAGTACTCACTGTTACGACAC-3′; wsv279, 5′-TTGCTGCAACAATA TTCACAGTGAGGAAACATCCC-3′ and 5′-CTAAGGGATGTTTCCTCACTGTG AATATTG-3′; wsv309, 5′-CAATCTCTGTCCGTACAGTGAGGTACATCAAA-3′ and 5′-GAGGTGTTTGATGTACCTCACTGTACGGACAGAG-3′; wsv361, 5′-TCA CAAGCTCTCTGCACAGTGAGCCGACACT-3′ and 5′-GTATCCAGTGTCGGCTC ACTGTGCAGAGAGC-3′). All the recombinant plasmids were confirmed by sequencing.

Insect High Five cells (Invitrogen) were cultured at 27°C in Express Five serum-free medium (Invitrogen) containing L-glutamine (Invitrogen). When the cells cultured in 96-well plate were at about 70% confluence, they were co-transfected with 50 nM miRNA and 200 ng EGFP-3′ UTR or EGFP-3′ UTR-mutant plasmid using Cellfectin II transfection reagent (Invitrogen) according to the manufacturer′s instructions. Six hours later, fresh medium was added to the cells and the cells were cultured for an additional 48 h. Subsequently, the fluorescent cells were photographed under a fluorescence microscope (Nikon Instruments, Melville, New York, USA) and the green fluorescence of the cells was monitored with a Flex Station II microplate reader (Molecular Devices, Foster City, CA, USA) at 490/510 nm of excitation/emission. The fluorescence values were corrected by subtracting the autofluorescence of untreated cells. All the experiments were repeated biologically three times.

### miRNA overexpression and RNAi assay in shrimp *in vivo*

Shrimp were simultaneously injected with WSSV virions (10^4^ copies/shrimp) and/or 30 μg of a synthesized siRNA (*wsv140*-siRNA, 5′-GCGAGCGAUGACGUCA UUU-3′; *wsv279*-siRNA, 5′-GCAAUUCCCUUAGCGGAAA-3′; *wsv309*-siRNA, 5′- CCGGACAAACUCCCUGUAA-3′; *wsv361*-siRNA, 5′-GGGAAUAUUUGUGACC GAA-3′) or mja-miR-35. As controls, PBS alone, WSSV alone (10^4^ copies/shrimp) and 30 μg of mja-miR-35-scrambled or siRNA-scrambled (5′-UUCUCCGAACGUG UCACGUUU-3′) were included in the injections. The miRNAs and siRNAs were synthesized using *in vitro* transcription T7 kit (Takara) according to the manufacturer′s instructions. At different time after injection, the shrimp hemocytes were collected for later use.

### Dual-luciferase reporter assay

To investigate the direct interaction between mja-miR-35 and its predicted target *CHI3L1* gene, a dual-luciferase reporter assay was conducted. The wild-type *CHI3L1* 3′ UTR was cloned into pmir-GLO vector (Promega, Madison, WI, USA) using sequence-specific primers (5′-GAGCTCTTCTGCACACAGCACGGGG-3′ and 5′-C TCGAGCACTGTTAAGCTCTTGTAC-3′), generating *CHI3L1* 3′ UTR construct. In this construct, the binding sites of mja-miR-35 seed sequence (oligonucleotides CACAGT) in *CHI3L1* 3′ UTR were mutated to oligonucleotides TCTCAG, yielding *CHI3L1* 3′ UTR mutant. The 293T cells at a density of 4 × 10^4^ cells/well were co-transfected with miRNA (100 nM) and pmir-GLO vector using Lipofectamine 2,000 reagent (Invitrogen) according to the manufacturer's procedures. Six hours later, fresh medium was added to the cells and the cells were cultured for an additional 48 h. Subsequently firefly luciferase and Renilla luciferase activities were examined using the Dual-Glo® Luciferase Assay System according the manufacturer's protocol (Promega). The level of firefly luciferase activity was normalized to that of Renilla luciferase.

### Boyden chamber cell-migration assay

Breast cancer cell migration was evaluated using 24-well Boyden chambers (Corning) with 8-μm pore-size inserts. MDA-MB-231 cells suspended in FBS-free medium were plated onto the inserts of the upper chamber at a density of 2 × 10^4^ cells/well. Then M2 macrophages were added to the lower chamber. The cells were co-cultured at 37°C. At 12 h after co-culture, MDA-MB-231 cells were fixed with 4% paraformaldehyde (Sigma) and stained with 0.005% crystal violet (Beyotime). The number of migrated cells was counted using a phase-contrast microscope.

### Adhesion assay

The ability of MDA-MB-231 cells to adhere to human fibronectin was examined in a 24-well plate. A 0.5-mL aliquot of culture medium containing 10–20 μg/ml of fibronectin (BD Biosciences) was added to each well and incubated at 4°C overnight. After removal of the supernatant, the plate was blocked for 30 min at 37°C with culture medium containing 10% BSA (Sigma) (w/v). The plate was rinsed three times with PBS. Subsequently, co-cultured MDA-MB-231 cells which were resuspended in serum-free culture medium were transferred to the plate (1.2 × 10^5^ cells/well) and allowed to adhere for 30 min at 37°C. The non-adherent cells were removed and the wells were gently washed twice with PBS. The adherent cells were stained using 0.005% crystal violet (Beyotime) and the numbers of cells per field of view were counted under a microscope.

### Invasion assay

To evaluate the breast cancer cell invasion, the experiments were conducted using 24-well invasion chambers with 8-μm pore-size inserts that were coated with Matrigel (Corning). MDA-MB-231 cells (1.5 × 10^5^ cells/well) were plated on the inserts and were co-cultured with M2 macrophages for 24 h at 37°C. The cells that invaded across the inserts were stained with 0.005% crystal violet (Beyotime), followed by counting cells per field of view under a phase-contrast microscope.

### Assessment of breast cancer metastasis *in vivo*

The breast cancer metastasis assay was conducted in mice. Female BALB/c nude mice (5~6 weeks old) were used in this study. Breast cancer cells (2 × 10^5^ MDA-MB-231 cells) stably expressing the firefly luciferase reporter were resuspended in 100 μl of PBS. Then the mixture was intravenously injected into mice. One day later, 50 μg miRNA dissolved in 100 μl PBS or PBS alone was injected into the mice via the tail vain. PBS or miRNA was injected twice a week over a 5-week period. To evaluate the effects of mja-miR-35 from shrimp fed mja-miR-35-expressing bacteria *in vivo*, the mice injected with cancer cells were fed with the muscle of shrimp fed mja-miR-35-expressing bacteria or bacteria alone daily. For *in vivo* imaging, the mice were intraperitoneal injected with D-luciferin at a dosage of 150 mg/kg in PBS. The bioluminescence signal of lung metastasis was monitored every 2 weeks via an IVIS Spectrum Imaging System (Perkin Elmer, USA). Bioluminescence analysis was performed using Living Image software version 4.5 (Perkin Elmer).

### Ethics statement

The animal experiments were approved by the ethical board of Zhejiang University Experimental Animals Ethics Committee (Permit Number: ZJU201308-1-10-072) and were performed according to Guide for the Care and Use of Laboratory Animals of the U.S. National Institutes of Health. All efforts were made to minimize the number of animals and their suffering.

### Prokaryotic expression of mja-miR-35 in bacteria

The sense and antisense strands of mja-miR-35 were annealed in annealing buffer (100 mM Tris-HCl, 500 mM NaCl, 10 mM EDTA, pH 8.0). The annealed product was cloned into LITMUS 38i vector (New England Biolabs, UK). Then the recombinant plasmid was transformed into *Escherichia coli* HT115 (DE3) strain. When OD_600_ of the bacteria reached 0.6, IPTG at a final concentration of 1 mM was added to induce the expression of mja-miR-35. After culture for further 4 h, the bacteria were collected for later use.

### Administration of shrimp feed expressing mja-miR-35

The bacteria expressing mja-miR-35 were heat-inactivated at 60° for 1 h. After centrifugation at 10,000 × g for 10 min, the bacteria were mixed with shrimp feed at a concentration of 3 × 10^12^ CFU/g feed. The WSSV-infected shrimp were fed with shrimp feed daily. At different times after feeding, the shrimp were collected for later use.

### Statistical analysis

Numerical data were analyzed with a one-way analysis of variance using SPSS statistical software. The statistical significance between different treatments was evaluated using Student's *t*-test. All experiments were biologically repeated three times.

## Results

### Suppression of breast cancer cell metastasis and shrimp virus infection by shrimp mja-miR-35

**Figure 1:**
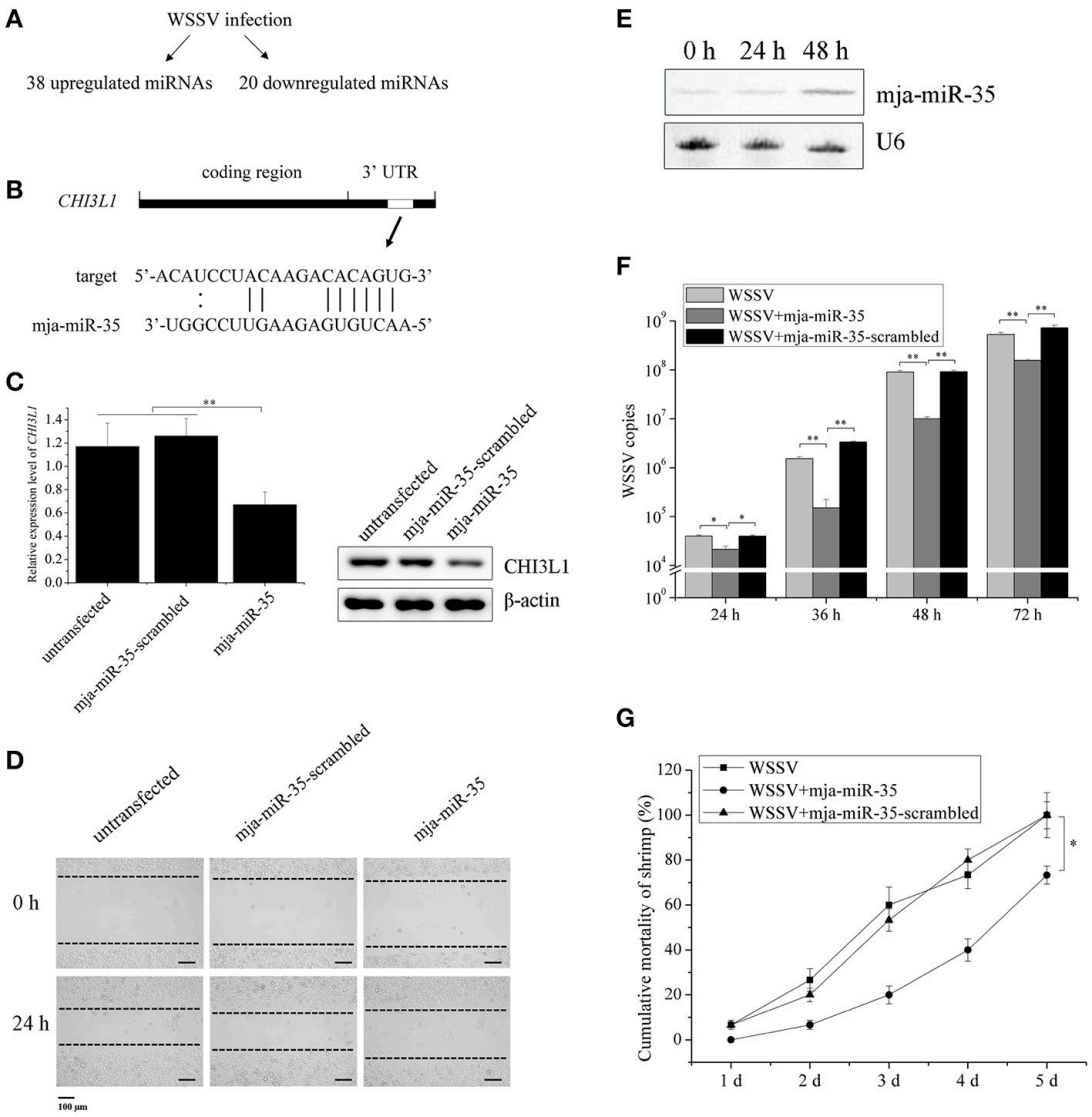
Suppression of breast cancer cell metastasis and shrimp virus infection by shrimp mja-miR-35. **(A)** The upregulated and downregulated miRNAs in shrimp in response to WSSV infection. **(B)** The prediction of miRNAs targeting human *CHI3L1* gene. As predicted, the *CHI3L1* 3′UTR was targeted by mja-miR-35 from 38 upregulated miRNAs. The seed sequence of mja-miR-35 was underlined. **(C)** Effects of mja-miR-35 on CHI3L1 expression in M2 macrophages. M2 macrophages were transfected with the synthesized mja-miR-35 or mja-miR-35-scrambled. At 24 h after transfection, the CHI3L1 transcript level was determined using quantitative real-time PCR. The secreted CHI3L1 protein level of M2 macrophages was examined using Western blotting. GAPDH and β-actin served as loading controls. **(D)** The effects of mja-miR-35 expression in M2 macrophages on breast cancer cell migration. M2 macrophages were transfected with the synthesized mja-miR-35 and co-cultured with breast cancer cells MDA-MB-231. As a control, mja-miR-35-scrambled was used. A linear wound was created in the confluent monolayer of MDA-MB-231 cells. At 24 h after co-culture, the cancer cells were examined using microscopy. **(E)** Expression of mja-miR-35 in shrimp in response to WSSV infection. At different time points post-infection, mja-miR-35 was detected in hemocytes of WSSV-infected shrimp by Northern blotting. U6 was used as a loading control. **(F)** The influence of mja-miR-35 on WSSV infection. The synthesized mja-miR-35 was injected into WSSV-infected shrimp. At different time post-infection, the shrimp were subjected to quantitative real-time PCR to quantify the WSSV copies. As controls, WSSV alone and mja-miR-35-scrambled were included in the injections. **(G)** The effects of mja-miR-35 on the mortality of WSSV-infected shrimp. The shrimp were simultaneously injected with WSSV and mja-miR-35. WSSV alone and mja-miR-35-scrambled were included in the injections as controls. At different time after injection, the cumulative mortality of shrimp was monitored. Data presented were representatives of triplicate assays (*p < 0.05; **p < 0.01).

Based on our previous studies (22, 23), WSSV infection resulted in significant upregulation of 38 miRNAs and downregulation of 20 miRNAs in shrimp (Figure 1A), suggesting that these miRNAs might synchronously possess antiviral activity in shrimp and anti-tumor activity in humans. Our previous study revealed that M2 macrophage-secreted CHI3L1 protein promoted breast cancer metastasis (21). In this context, the upregulated miRNAs in shrimp in response to WSSV challenge might be good candidates for discovering anti-tumor miRNAs by targeting *CHI3L1* gene. The target gene prediction results showed that a shrimp-specific miRNA (mja-miR-35) from the 38 upregulated miRNAs could target human *CHI3L1* gene (Figure 1B). To explore the *in vivo* interaction between mja-miR-35 and the *CHI3L1* gene in M2 macrophages, the synthesized mja-miR-35 was transfected into M2 macrophages and then the CHI3L1 expression level was examined. The results indicated that the shrimp mja-miR-35 expression in human M2 macrophages led to significantly reduced transcript level and secretion level of CHI3L1 protein (Figure 1C), indicating that mja-miR-35 was interacted with human *CHI3L1 in vivo*.

To investigate the effects of mja-miR-35 on cancer cell migration, M2 macrophages were transfected with the synthesized shrimp mja-miR-35. Twenty-four hours later, M2 macrophages were co-cultured with MDA-MB-231 cells and then subjected to wound-healing assays. The results showed that the mja-miR-35 expression in M2 macrophages significantly inhibited breast cancer cell migration (Figure 1D), indicating that mja-miR-35 could suppress breast cancer metastasis by targeting *CHI3L1* gene.

To evaluate the influence of mja-miR-35 on shrimp antiviral immunity, shrimp were challenged with WSSV, followed by the detection of mja-miR-35 expression. Northern blotting data revealed that WSSV infection led to a significant increase of mja-miR-35 expression level at 48 h post-infection (Figure 1E), indicating the involvement of mja-miR-35 in WSSV infection. The mja-miR-35 overexpression significantly decreased the WSSV copies in shrimp and the mortality of WSSV-infected shrimp compared with the controls (Figures 1F,G). At the same time, the co-injection of WSSV and mja-miR-35-scrambled into shrimp yielded similar results to those of the positive control (WSSV alone) (Figures 1F,G), showing that mja-miR-35 was not cytotoxic to shrimp. These data indicated that mja-miR-35 possessed antiviral activity in shrimp.

Taken together, the findings presented that shrimp mja-miR-35 could suppress cell metastasis of breast cancer and virus infection of shrimp.

### Antiviral mechanism of mja-miR-35 in shrimp

**Figure 2:**
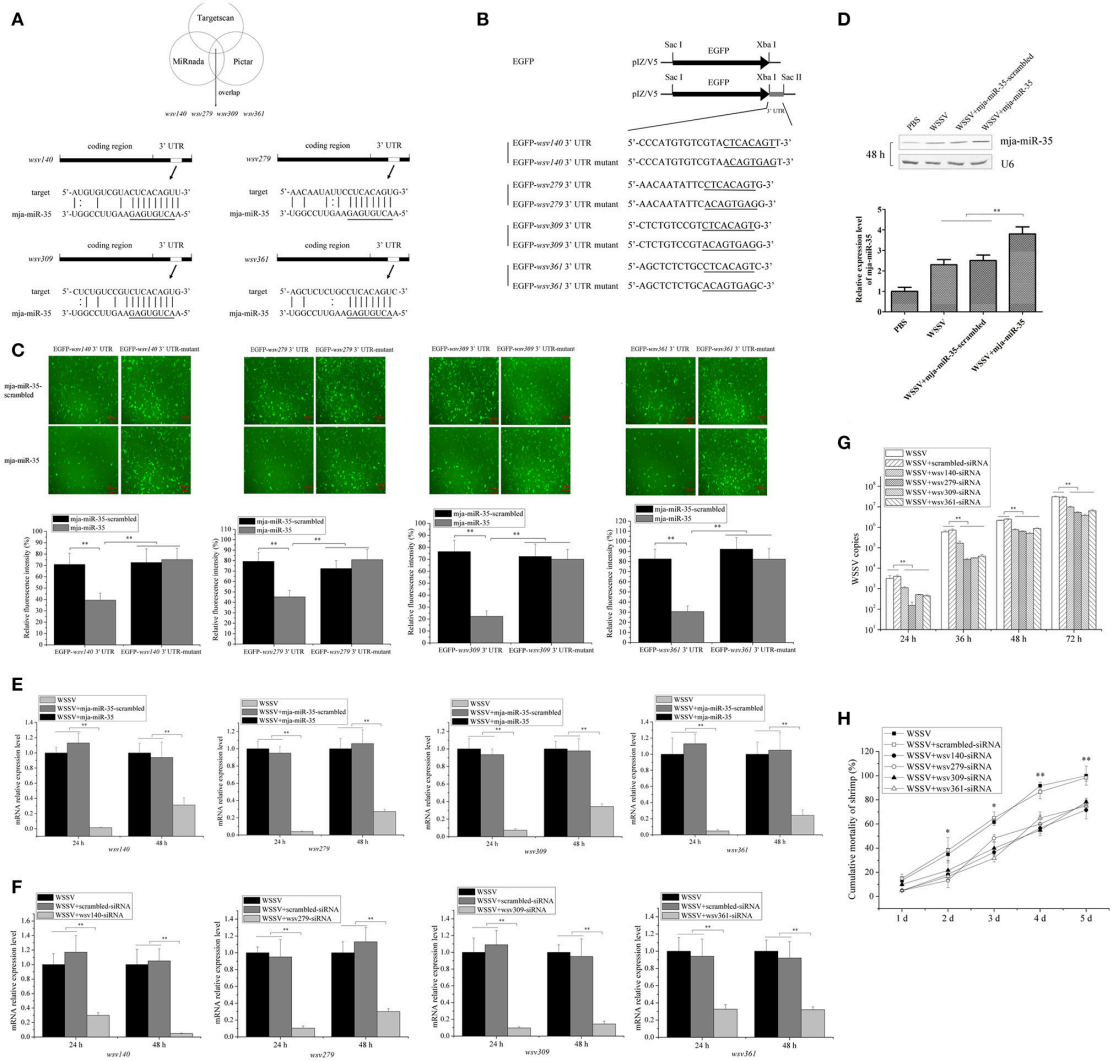
Antiviral mechanism of mja-miR-35 in shrimp. **(A)** Predicted target genes of mja-miR-35. As predicted, the 3′ UTRs of *wsv140, wsv279, wsv309*, and *wsv361* genes were targeted by mja-miR-35. The seed sequence of mja-miR-35 was underlined. **(B)** Constructs of the wild-type and mutated 3′ UTRs of the viral gene. The sequences complementary to the seed sequence of mja-miR-35 or the mutated seed sequences were underlined. **(C)** Interaction between mja-miR-35 and the viral *wsv140, wsv279, wsv309*, and *wsv361* genes in insect cells. The insect High Five cells were co-transfected with mja-miR-35 and different plasmids (EGFP-3′ UTR or EGFP-3′ UTR-mutant). As a control, mja-miR-35-scrambled was included in the transfection. At 48 h after transfection, fluorescent images (upper panel) and fluorescence intensities (lower panel) were obtained. Lane headings indicated the plasmids used. The miRNAs for transfections were shown on the left. Scale bar, 10 μm. **(D)** Overexpression of mja-miR-35 in shrimp. Shrimp were co-injected with WSSV and mja-miR-35 or mja-miR-35-scrambled. PBS and WSSV alone were used as controls. At 48 h after injection, shrimp hemocytes were subjected to Northern blotting with mja-miR-35-specific probe. U6 was used as a loading control. **(E)** Effects of mja-miR-35 overexpression on viral gene expression *in vivo*. WSSV- infected shrimp were injected with mja-miR-35 or scrambled-miRNA. At 24 h and 48 h after injection, the shrimp hemocytes were subjected to quantitative real-time PCR to examine the expression levels of *wsv140, wsv279, wsv309*, and *wsv361* genes. **(F)** Silencing of virus gene expression in shrimp. Sequence-specific siRNA was injected into WSSV-infected shrimp to knock down the expression of virus genes. At different time points after injection, the shrimp hemocytes were analyzed using quantitative real-time PCR. WSSV alone and scrambled-siRNA were used as controls. **(G)** Influence of virus gene silencing on WSSV replication. Gene-specific siRNA or scrambled-siRNA was injected into WSSV-infected shrimp. At different time points after infection, the hemocytes of shrimp were subjected to quantitative real-time PCR analysis to detect WSSV copies. WSSV alone was used as a positive control. **(H)** Effects of virus gene silencing on the mortality of WSSV-infected shrimp. The shrimp were injected with different siRNAs. The shrimp mortality was monitored daily. WSSV alone was used as a control. In all panels, the statistically significant difference between treatments was indicated with asterisks (*p < 0.05; **p < 0.01).

To reveal the mja-miR-35-mediated pathways in shrimp, the target genes of mja-miR-35 were predicted. Based on target prediction using the TargetScan, Pictar, and miRanda algorithms, it was found that four WSSV genes (*wsv140, wsv279, wsv309*, and *wsv361*) might be the targets of mja-miR-35 (Figure 2A). To verify the interaction between mja-miR-35 and its targets, EGFP and the WSSV gene 3′ UTR or 3′ UTR mutant were constructed into pIZ/V5 vector (Figure 2B). The synthesized mja-miR-35 and the plasmid EGFP-3′ UTR were co-transfected into insect High Five cells. The results demonstrated that the fluorescence intensity in the cells co-transfected with mja-miR-35 and wild-type 3′ UTR of *wsv140, wsv279, wsv309*, or *wsv361* genes was significantly decreased compared with that of the controls (Figure 2C), indicating that mja-miR-35 directly targeted the 3′ UTR of *wsv140, wsv279, wsv309*, and *wsv361* genes.

To further evaluate the *in vivo* interaction between mja-miR-35 and the viral genes in shrimp, the synthesized mja-miR-35 was injected into WSSV-infected shrimp, followed by detections of viral genes' expressions. Northern blots indicated that mja-miR-35 was overexpressed in shrimp compared with the control (Figure 2D). The mja-miR-35 overexpression led to significant decreases of *wsv140, wsv279, wsv309* and *wsv361* mRNAs in WSSV-infected shrimp compared with the controls (Figure 2E), indicating the interactions between mja-miR-35 and viral *wsv140, wsv279, wsv309*, and *wsv361* genes in shrimp *in vivo*.

In an attempt to reveal the roles of *wsv140, wsv279, wsv309*, and *wsv361* genes in WSSV infection, the expressions of these four genes were silenced by gene-specific siRNAs in shrimp, respectively. The results showed that the expression levels of *wsv140, wsv279, wsv309*, and *wsv361* genes were significantly decreased in WSSV-infected shrimp, while the scrambled-siRNA had no influence on the virus gene expression (Figure 2F), showing that the viral gene expression was specifically silenced. The silencing of *wsv140, wsv279, wsv309*, or *wsv361* resulted in a significant decrease of WSSV copies and a significant decrease of WSSV-infected shrimp mortality compared with the scrambled-siRNA control (Figures 2G,H), showing the requirement of *wsv140, wsv279, wsv309*, and *wsv361* genes for WSSV infection.

The above findings indicated that mja-miR-35 could target viral genes (*wsv140, wsv279, wsv309*, and *wsv361*) to suppress virus infection in shrimp *in vivo*.

### Underlying mechanism of mja-miR-35-mediated suppression of breast cancer cell metastasis *in vivo*

**Figure 3:**
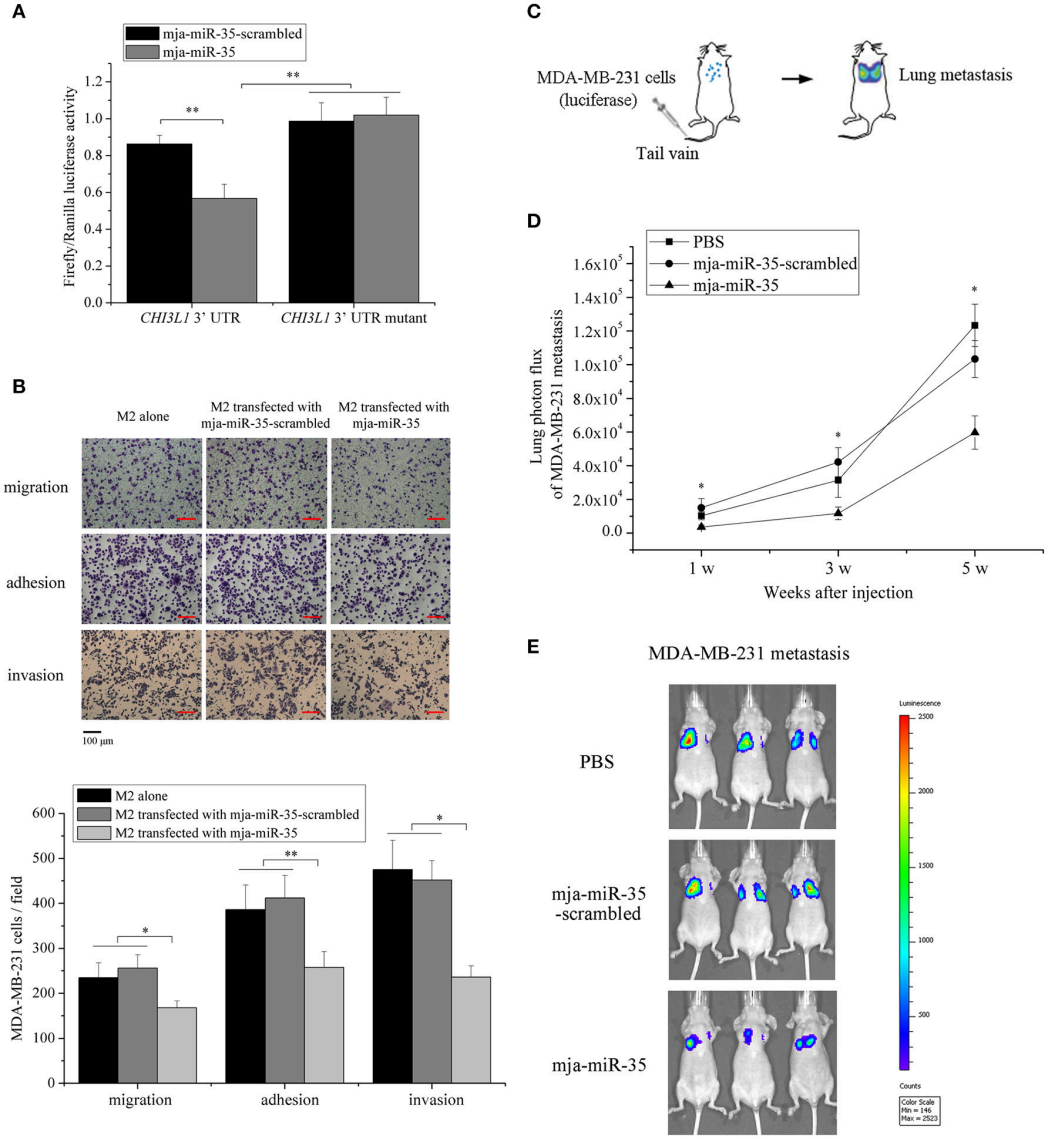
Underlying mechanism of mja-miR-35-mediated suppression of breast cancer cell metastasis. **(A)** The direct interaction between mja-miR-35 and *CHI3L1* 3′ UTR. The 293T cells were co-transfected with mja-miR-35 and pmir-GLO vector fused with *CHI3L1* 3′ UTR. Firefly and Renilla luciferase activities were determined. As controls, mja-miR-35-scrambled and *CHI3L1* 3′ UTR mutant were included in the co-transfections (**p < 0.01). **(B)** The impact of mja-miR-35 expression in M2 macrophages on breast cancer cell metastasis. MDA-MB-231 cells were co-cultured with mja-miR-35-transfected M2 macrophages, followed by cell migration, adhesion and invasion assays (*p < 0.05; **p < 0.01). Scale bar, 100 μm. **(C)** Schematic diagram of MDA-MB-231 cell metastasis to lung following intravenous injection in mouse. **(D)** The effects of mja-miR-35 on breast cancer cell metastasis *in vivo*. MDA-MB-231 cells were intravenously injected into BALB/c nude mice. One day later, mja-miR-35, mja-miR-35-scrambled or PBS was injected into the mice. Lung metastasis was monitored every 2 weeks (*p < 0.05). **(E)** Representative images of MDA-MB-231 cell metastasis in mice at week 5.

To explore the underlying mechanism of mja-miR-35-mediated suppression of breast cancer cell metastasis, the interaction between mja-miR-35 and *CHI3L1* gene was characterized. The results showed that when mja-miR-35 was co-transfected with pmir-GLO dual-luciferase construct containing wild-type *CHI3L1* 3′ UTR into cells, the luciferase activity of cells was significantly decreased compared with the controls (Figure 3A), indicating that mja-miR-35 was directly interacted with *CHI3L1* 3′ UTR.

To evaluate the influence of mja-miR-35 on breast cancer cell metastasis, MDA-MB-231 cells were co-cultured with mja-miR-35-transfected M2 macrophages and then subjected to Boyden chamber assay, adhesion and invasion assays. The results indicated that the number of migrated cancer cells was significantly reduced by co-culturing with mja-miR-35-transfected M2 macrophages compared with the controls (untreated or mja-miR-35-scrambled-transfected M2 macrophages) (Figure 3B). The adhesion and invasiveness activities of cancer cells were remarkably decreased when cancer cells were co-cultured with mja-miR-35-transfected M2 macrophages (Figure 3B). These findings revealed that mja-miR-35 could inhibit breast cancer metastasis by directly targeting *CHI3L1* gene of M2 macrophages.

To further explore the effects of mja-miR-35 on breast cancer metastasis *in vivo*, MDA-MB-231 cells with a stably integrated luciferase reporter were intravenously injected into BALB/c nude mice to establish a model for breast cancer lung metastasis assays (Figure 3C). The results indicated that the mice injected with mja-miR-35 had much lower lung metastasis luciferase signals than those of controls (Figures 3D,E), showing that mja-miR-35 could inhibit breast cancer metastasis *in vivo*.

Taken the above findings together, shrimp mja-miR-35 could suppress breast cancer metastasis *in vivo* by targeting the human *CHI3L1* gene.

### Antiviral activity of shrimp FED mja-miR-35-expressing bacteria *in vivo*

**Figure 4:**
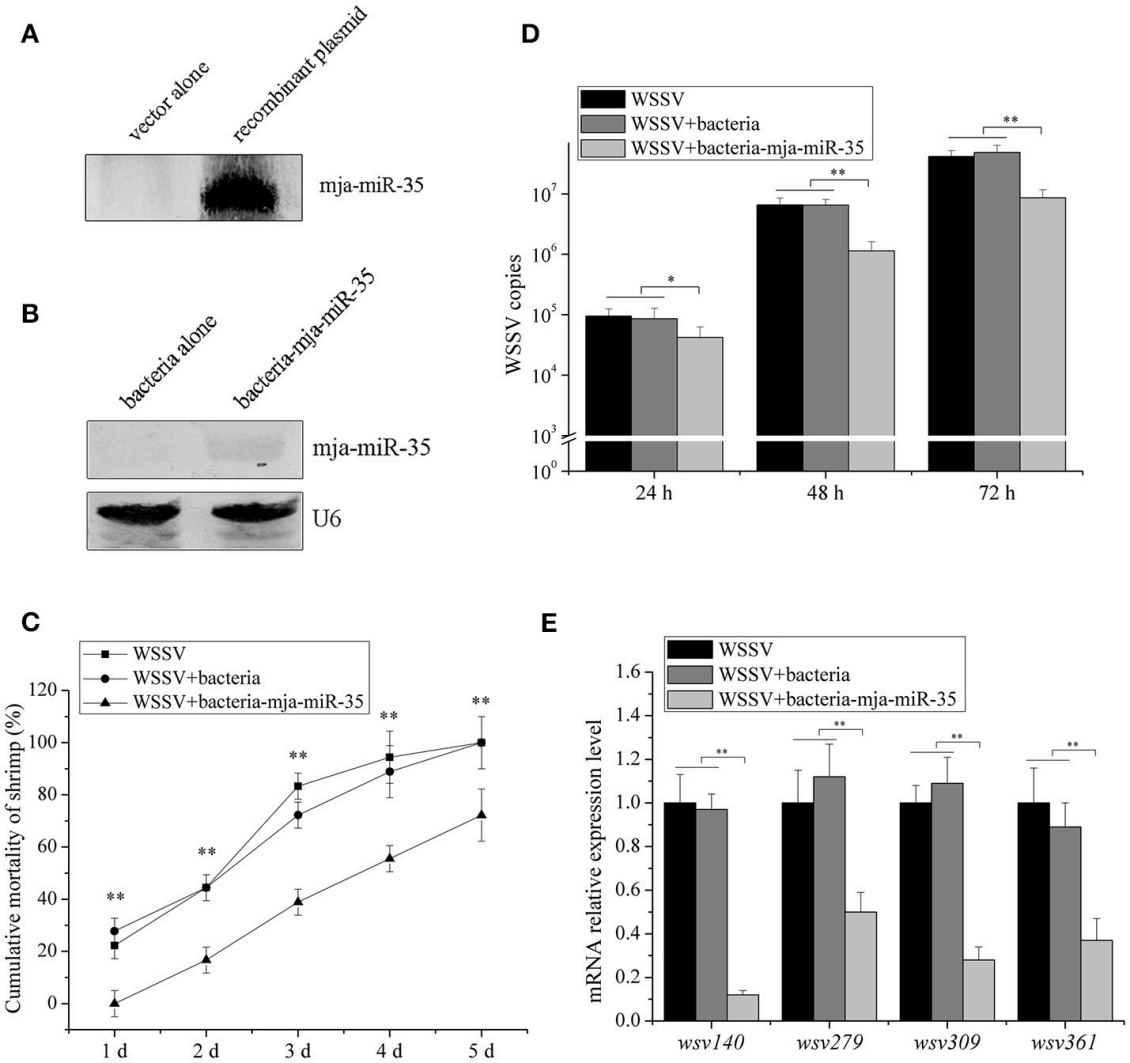
Antiviral activity of shrimp fed mja-miR-35-expressing bacteria *in vivo*. **(A)** Expression of mature mja-miR-35 in bacteria. LITMUS 38i vector (vector alone) or LITMUS 38i-miR-35 expressing mja-miR-35 (recombinant plasmid) was transformed into *Escherichia coli* HT115 cells. After IPTG induction, Northern blotting was conducted to detect mja-miR-35. **(B)** Detection of mature mja-miR-35 in shrimp fed with bacteria expressing mja-miR-35. The bacteria expressing mja-miR-35 were mixed with shrimp feed and then the shrimp were fed with the mixed feed daily. At 48 h after feeding, the expression level of mja-miR-35 was detected by Northern blotting. U6 served as a loading control. **(C)** Influence of mja-miR-35 in shrimp fed with bacteria-mja-miR-35 on WSSV replication. The WSSV-infected shrimp were fed shrimp feed containing bacteria-mja-miR-35 or bacteria-mja-miR-35-scrambled daily. At different post-infection times, WSSV copy number was quantified by real-time PCR. WSSV alone was used as a positive control. **(D)** Effects of mja-miR-35 in shrimp fed with bacteria-mja-miR-35 on shrimp cumulative mortality. The shrimp were treated with WSSV and fed shrimp feed containing bacteria- mja-miR-35 or bacteria- mja-miR-35-scrambled. The shrimp cumulative mortality was examined every day. The treatments were shown at the top. **(E)** Impact of mja-miR-35 in shrimp fed with bacteria-mja-miR-35 on the expression of mja-miR-35 target viral genes. Shrimp were fed shrimp feed+bacteria-miR-34/bacteria-miR-34-scrambled daily. The shrimp were then infected with WSSV. WSSV alone was used as a control. At 48 h post-infection, the mRNA levels of viral genes were evaluated using quantitative real-time PCR. Statistically significant differences between treatments were indicated with asterisks (*p < 0.05; **p < 0.01).

To explore the influence of feed containing shrimp mja-miR-35 on shrimp antiviral activity, mja-miR-35 was expressed in bacteria. Northern blot analysis indicated that mature mja-miR-35 was expressed in IPTG-induced recombinant bacteria (Figure 4A). The IPTG-induced recombinant bacteria expressing mja-miR-35 (bacteria-mja-miR-35) were mixed with shrimp feed for feeding shrimp. The results showed that the expression level of mja-miR-35 in the shrimp fed with bacteria-mja-miR-35 was significantly upregulated compared with the control (Figure 4B), indicating that mja-miR-35 in feed was absorbed by shrimp. The continuously high expression level of mja-miR-35 in shrimp led to a significant decrease in WSSV copy number (Figure 4C) and virus-infected shrimp mortality (Figure 4D). However, the WSSV copy number and the mortality of shrimp fed the shrimp feed containing bacteria alone were consistent with the control WSSV alone (Figures 4C,D). These data revealed that the elevated expression level of mature mja-miR-35 in shrimp fed the bacteria expressing mja-miR-35 promoted the antiviral immune response of shrimp *in vivo*.

To investigate the impact of mja-miR-35 in shrimp fed bacteria-mja-miR-35 on the expression of mja-miR-35 target viral genes, the expression of *wsv140, wsv279, wsv309*, and *wsv361* in shrimp after feeding mja-miR-35 was examined. Quantitative real-time PCR revealed that the overexpression of mja-miR-35 downregulated the expression of mja-miR-35 target genes (Figure 4E).

These findings suggested that mja-miR-35 in shrimp fed bacteria containing mja-miR-35 inhibited virus infection by targeting viral *wsv140, wsv279, wsv309*, and *wsv361* genes in shrimp *in vivo*.

### Inhibition of breast cancer lung metastasis by mja-miR-35 from shrimp fed mja-miR-35-expressing bacteria *in vivo*

**Figure 5:**
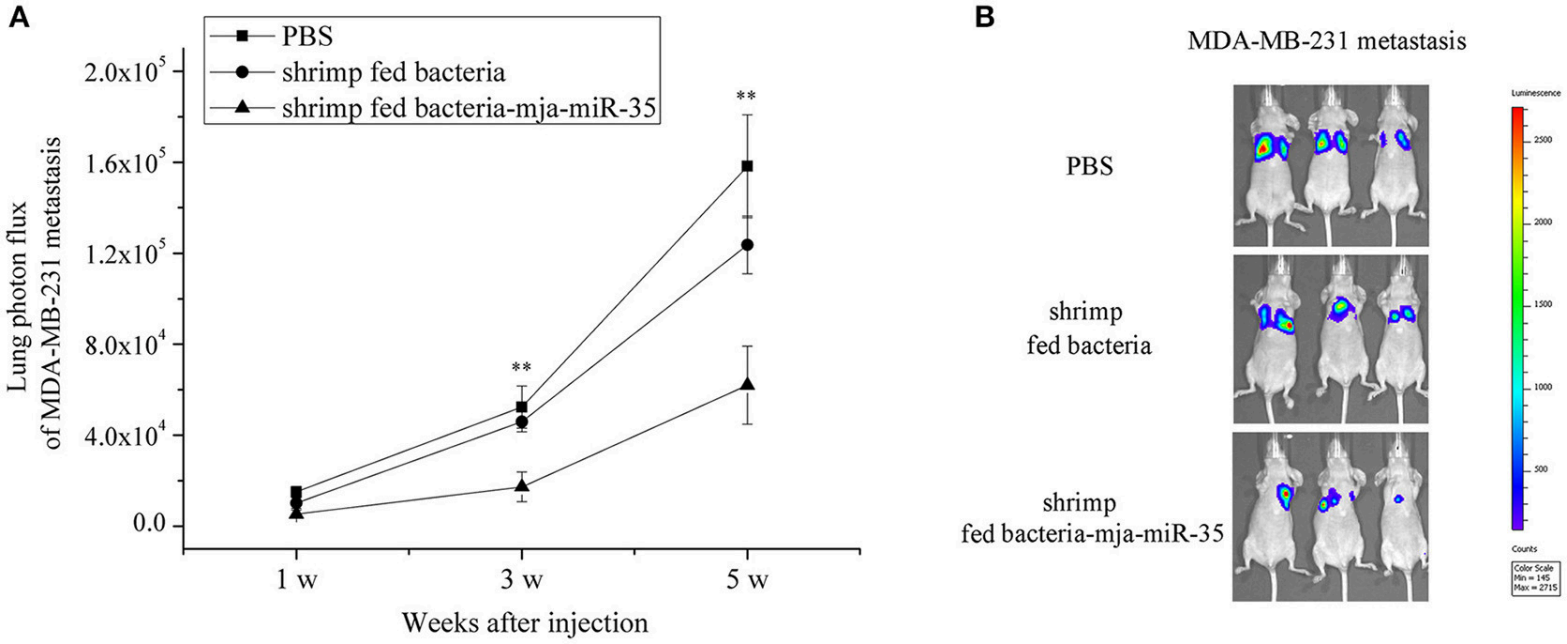
Inhibition of breast cancer lung metastasis by mja-miR-35 from shrimp fed mja-miR-35-expressing bacteria *in vivo*. **(A)** Effects of mja-miR-35 from shrimp fed mja-miR-35-expressing bacteria on breast cancer metastasis. MDA-MB-231 cells were intravenously injected into BALB/c nude mice (*n* = 5/treatment). One day later, the mice were fed with shrimp fed mja-miR-35-expressing bacteria or bacteria alone daily. PBS was served as a control. Lung metastasis was monitored via *in vivo* quantitative luciferase bioluminescence imaging every 2 weeks. The numbers on the horizontal axis indicated the weeks after the injection of MDA-MB-231 cells. **(B)** Representative images of MDA-MB-231 cell metastasis in mice at week 5. Statistically significant differences between treatments were indicated with asterisks (**p < 0.01).

To further illustrate the anti-tumor effects of antiviral mja-miR-35 from shrimp fed mja-miR-35-expressing bacteria *in vivo*, MDA-MB-231 cells were intravenously injected into BALB/c nude mice and the mice were fed with the muscle of shrimp fed bacteria-mja-miR-35. The results of quantitative bioluminescence imaging analysis showed that the feeding with the muscle of shrimp fed mja-miR-35-expressing bacteria significantly decreased the lung metastasis of cancer cells in mice compared with the controls (Figure 5A). At the 5th week, the bioluminescence signals of mice fed with the muscle of shrimp fed mja-miR-35-expressing bacteria were much lower than those of PBS-treated mice and mice fed with the muscle of shrimp fed bacteria alone (Figure 5B). These findings revealed that mja-miR-35 from shrimp fed with mja-miR-35-expressing bacteria could suppress breast cancer metastasis by targeting the human *CHI3L1* gene *in vivo*.

## Discussion

The existence of life is finely governed by few but essential life processes such as duplication of DNA, RNA synthesis from DNA and protein synthesis from RNA. As a family of small noncoding RNAs, miRNAs can control the contents of cellular transcribed RNAs and subsequently influence protein levels for numerous biological processes, including tissue and organ development, cell signaling, biochemical pathways (24). During virus-host interactions, miRNAs play key roles as important post-transcriptional regulators of gene expressions (22, 25, 26). Viruses complete their life cycles in host cells, thus leading to the metabolic disorder of host cells (27). Hence, to deal with the metabolic disorder caused by virus infection, the regulation of gene expressions by miRNAs becomes very important for hosts to defend against virus infection. As well-known, cancer is a typical example of a common human disease with pathological metabolic perturbations. As a hallmark of cancer, altered cellular metabolism contributes to malignant transformation and the initiation, growth and metastasis of tumors (28, 29). In this context, miRNAs with antiviral activity may play vital roles in restoring the metabolic disorder of tumor cells. In the present study, the findings revealed that shrimp mja-miR-35, an antiviral miRNA in shrimp, could suppress the metastasis of human breast cancer. As reported, an individual miRNA can target different genes (22, 23), suggesting that a miRNA had the capacity to target various genes from various species. Our study showed that shrimp mja-miR-35 inhibited virus infection of shrimp by targeting virus genes (*wsv147, wsv279, wsv309*, and *wsv361*) and took an antitumor effect of human breast cancer by targeting CHI3L1, an oncogenic gene in macrophages (21). It is reported that *CHI3L1*-knockout tumor bearing mice has a significant reduction in tumor volume and metastasis compared with the wild-type controls (30). After controlled cortical impact, the *CHI3L1* knockout mice demonstrate more severe neuropathology than their wild-type littermates (31). As reported, the CHI3L1 protein can be produced by various cell types, including differentiated macrophages, neutrophils, synovial cells, osteoblasts, chondrocytes, vascular smooth muscle cells, and tumor cells (32, 33). Among various cell types, M2 macrophages are primarily involved in tumor metastasis (21). In our previous study, it was revealed that the CHI3L1 protein was specifically secreted by M2 macrophages and was not expressed in MDA-MB-231 breast cancer cells (21). Thus, it might be concluded that mja-miR-35 inhibited breast cancer metastasis by silencing the *CHI3L1* gene expression. As reported, human miR-24 can target *CHI3L1* gene in monocytes, thereby modulating *S. aureus*-induced macrophage polarization, elevating anti-inflammatory factors expression in M2 macrophages and regulating osteomyelitis (34, 35). In the future investigation, the influence of mja-miR-35 on tumor cell growth and proliferation merited to be explored. In this context, our investigation indicated that the antiviral miRNAs from virus-challenged shrimp might be an important source of human antitumor drugs, which could function in a cross-phylum manner.

As an important seafood for humans, the production of shrimp has become a flourishing industry. However, the disease caused by WSSV infection is a limiting factor in shrimp farming. Shrimp defend themselves against pathogens by innate immunity, including the humoral and cellular immune responses (36). As reported, a number of shrimp miRNAs play essential roles in antiviral immunity of shrimp by targeting host or viral genes (37–42). Among these antiviral miRNAs, the results of this investigation revealed that shrimp mja-miR-35 possessed anti-tumor activity. The shrimp fed mja-miR-35-expressing feed could defend WSSV infection. At the same time, the mice fed mja-miR-35-overexpressing shrimp could suppress human breast cancer metastasis. It is reported that a plant miRNA is bio-available for human beings after early stage digestion via a simulated human digestion system (43). The shrimp mja-miR-35 might be absorbed by intestine and circulated in the plasma of shrimp or mouse. After transportation by high-density lipoprotein, the absorbed miRNA can be delivered to recipient cells with functional targeting capabilities (44). Therefore, the special feed expressing shrimp mja-miR-35 had the capacity of antivirus in shrimp and anti-tumor in humans, leading to the simultaneous controls of virus infection and tumorigenesis. Shrimp were potentially healthy foods against human breast cancer metastasis. Up to date, many miRNAs including shrimp miR-34 and mja-miR-35 are found to take great effects on tumorigenesis. The combination of these miRNAs might achieve a high suppression of tumor proliferation and metastasis. This concern merited to be further investigated.

## Author contributions

YC, SZ, JC, and XZ conceptualized the project and performed the investigations. YC, SZ, and XZ wrote the manuscript. XZ was responsible for overall supervision of the project.

### Conflict of interest statement

The authors declare that the research was conducted in the absence of any commercial or financial relationships that could be construed as a potential conflict of interest.
